# Co-Occurring Alteration of NOTCH and DDR Pathways Serves as Novel Predictor to Efficacious Immunotherapy in NSCLC

**DOI:** 10.3389/fonc.2021.659321

**Published:** 2021-04-22

**Authors:** Zhimin Zhang, Yanyan Gu, Xiaona Su, Jing Bai, Wei Guan, Jungang Ma, Jia Luo, Juan He, Bicheng Zhang, Mingying Geng, Xuefeng Xia, Yanfang Guan, Cheng Shen, Chuan Chen

**Affiliations:** ^1^ Cancer Center, Renmin Hospital of Wuhan University, Wuhan, China; ^2^ Department of Nutrition, Changzheng Hospital, Second Military Medical University, Shanghai, China; ^3^ Cancer Center, Daping Hospital, Army Medical University (Third Military Medical University), Chongqing, China; ^4^ Geneplus-Beijing Institute, Beijing, China; ^5^ Department of Thoracic Surgery, Daping Hospital, Army Medical University (Third Military Medical University), Chongqing, China

**Keywords:** NOTCH pathway, DDR pathway, co-occurring mutations, immunotherapy, predictive biomarker, non-small cell lung cancer

## Abstract

Although immune checkpoint inhibitors (ICIs) have shown remarkable benefit for treatment of advanced non-small lung cancer (NSCLC), only a minority of patients can achieve durable responses and the most patients produce an ultra-rapid progressive disease. Here, we collected the availably published datasets and mined the determinants of response to immunotherapy on pathway levels. One hundred six NSCLC patients treated with immunotherapy were combined from Rizvi et al. and Hellman et al. studies (whole exon sequencing). Two independent validation datasets consisted of the MSKCC cohort (targeted sequencing) and data by Anagnostou and colleagues (whole exon sequencing). The Cancer Genome Atlas (TCGA) somatic mutation and gene expression data were applied to explore the immunobiology features. In the first combined cohort, we detected NOTCH pathway altered in 71% patients with durable clinical benefit (DCB) while only 36% among no durable benefit (NDB) (p = 0.005). Compared to NDB group, co-occurrence of NOTCH and at least two DDR (co-DDR) pathway was discovered in DCB group and contributed to a prolonged progression-free survival (PFS) [22.1 *vs* 3.6 months, p < 0.0001, HR, 0.34, 95% confidence interval (CI), 0.2–0.59]. In two independent datasets, co-occurrence of NOTCH+/co-DDR+ was also validated to be a better immunotherapy efficacy [Cohort 2: 13 *vs* 6 months, p = 0.034, HR, 0.55, 95% CI, 0.31–0.96; Cohort 3: 21 *vs* 11 months, p = 0.067, HR, 0.45, 95% CI, 0.18–1.1]. By analyzing TCGA cohort, we found patients with coexisting NOTCH+/co-DDR+ pathway had a higher TMB, more infiltration of CD4+T cells. Overall, co-occurrence of NOTCH and co-DDR pathway reflect a better immunotherapy efficacy in advanced NSCLC. This genomic predictor show promise in stratifying patients that suit for immunotherapy for future clinical practice.

## Introduction

Immune checkpoint inhibitors (ICIs) has shown remarkable benefit for treatment of advanced non-small lung cancer (NSCLC) (1, 2). Nevertheless, only a limited patient population can generate durable responses after immunotherapy, while the majority of patients undergo inferior survival (3, 4). Therefore, there is an urgent need to stratify the patients who will benefit from ICIs.

A plethora of studies have shown biomarkers for predicting response to immunotherapy. The most heavily studied biomarker was programmed death-ligand 1 (PD-L1) expression correlated to efficacious immunotherapy (5–8), but subsequent trials proven a sizeable proportion of patients still achieve durable responses with PD-L1 negative (2, 9). Furthermore, some genomic markers have been reported for predicting the response to ICI, including tumor mutation burden (TMB) (10, 11), tumor neoantigen burden (TNB) (12, 13), and DNA repair alterations (14–16). Meanwhile, several studies explored the alterations on pathway level that correlates to ICI. Zhang et al. found NOTCH signaling related to better ICI efficacy (17). Wang et al. revealed co-mutations in DNA damage response (DDR) pathways could serve as predictors of response to ICB (18).

Due to the interactions and dependencies among different pathways, we supposed single pathway was not high enough to reflect the response of ICI. Therefore, we focused on the co-occurrent pathways to predict the superior survival outcomes after immunotherapy. Availably published datasets were collected from four studies. The first cohort was combined by data from Rizvi et al. and Hellman et al., which was used to identify the co-occurrent pathways correlated to ICI (15, 19). We further validated the co-occurrent pathways correlated to better efficacy after immunotherapy in two independent datasets (data from Anagnostou et al. and the MSKCC cohort) (20, 21). Using the somatic mutation and gene expression data from TCGA, we explored the immunobiology features about the co-occurrent pathway.

## Materials and Methods

### Datasets Used

We collected four publicly available datasets treated with immunotherapy (**Supplementary Table 1**). Thirty-four NSCLC patients sequenced by WES were downloaded from Rizvi et al. Another 75 patients with WES data were derived from Hellman et al. studies. Data from the two studies were merged as the first combined cohort. After excluding three patients lack of efficacy information, data from 106 patients was obtained. The second cohort was from Anagnostou et al., including 89 NSCLC patients treated with ICIs. Two patients were excluded because of lacking definitive response evaluation. For the third cohort, MSKCC cohort was downloaded from cBioPortal (http://www.cbioportal.org/study/summary?id=tmb_mskcc_2018). We also acquired the genome mutation from 1026 NSCLC patients and transcriptome expression data from 59 advanced NSCLC patients in TCGA.

### Pathway Alterations

Genes involved in 10 oncogenic signaling pathways and eight DDR pathways were listed in **Supplementary Table 2**. The 10 canonical oncogenic signaling pathways were downloaded from (22) and included: cell cycle, Hippo signaling, Myc signaling, Notch signaling, oxidative stress response/Nrf2, PI-3-Kinase signaling, receptor-tyrosine kinase (RTK)/RAS/MAP-Kinase signaling, TGFb signaling, p53 and b-catenin/Wnt signaling. Eight DDR pathways analyzed were: mismatch repair (MMR); base excision repair (BER); check point factors (CPF); Fanconi anemia (FA); homologous recombination repair (HRR); nucleotide excision repair (NER); non-homologous end-joining (NHEJ); and DNA translesion synthesis (TLS) (23). We defined pathway alteration as at least one gene in this pathway mutated. Co-DDR was defined as two or more DDR pathway altered. Co-occurring alteration of NOTCH and DDR pathway was referred as NOTCH+/co-DDR+.

### Immune Infiltration Analysis

SsGSEA was utilized to calculate the enrichment scores (ES) of immune cell types in the tumor microenvironment (24). Gene signatures of 28 immune cell types were downloaded from previous study (25). Tumors were further subclassified into different immune groups using the Euclidean distance and “ward.D” clustering. The expression levels of genes were first z-score normalized across all patients. Then we calculated the mean z-scores for each group and ranked in descending order. Based on the pre-ranked GSEA method, for each immune cell signature, we defined the q-value <10% and NES >0 as the enrichment, while the q-value <10% and NES <0 as the depletion.

### Statistical Analyses

We performed univariable and multivariable Cox regression analyses to identify potential predictors of survival. Survival curves were estimated with the Kaplan-Meier product-limit method and compared by log-rank test. Comparisons of TMB, TNB, and expression levels between different groups were used with the Wilcoxon rank-sum test or t test. Enrichment analysis of gene function was calculated by GSEA with Benjamini-Hochberg correction for multiple hypothesis testing (q < 0.05). All statistical analyses were performed in the R statistical environment version 3.6.2.

## Results

### Pathway Alterations in NSCLC Treated With Immunotherapy

**Figure 1:**
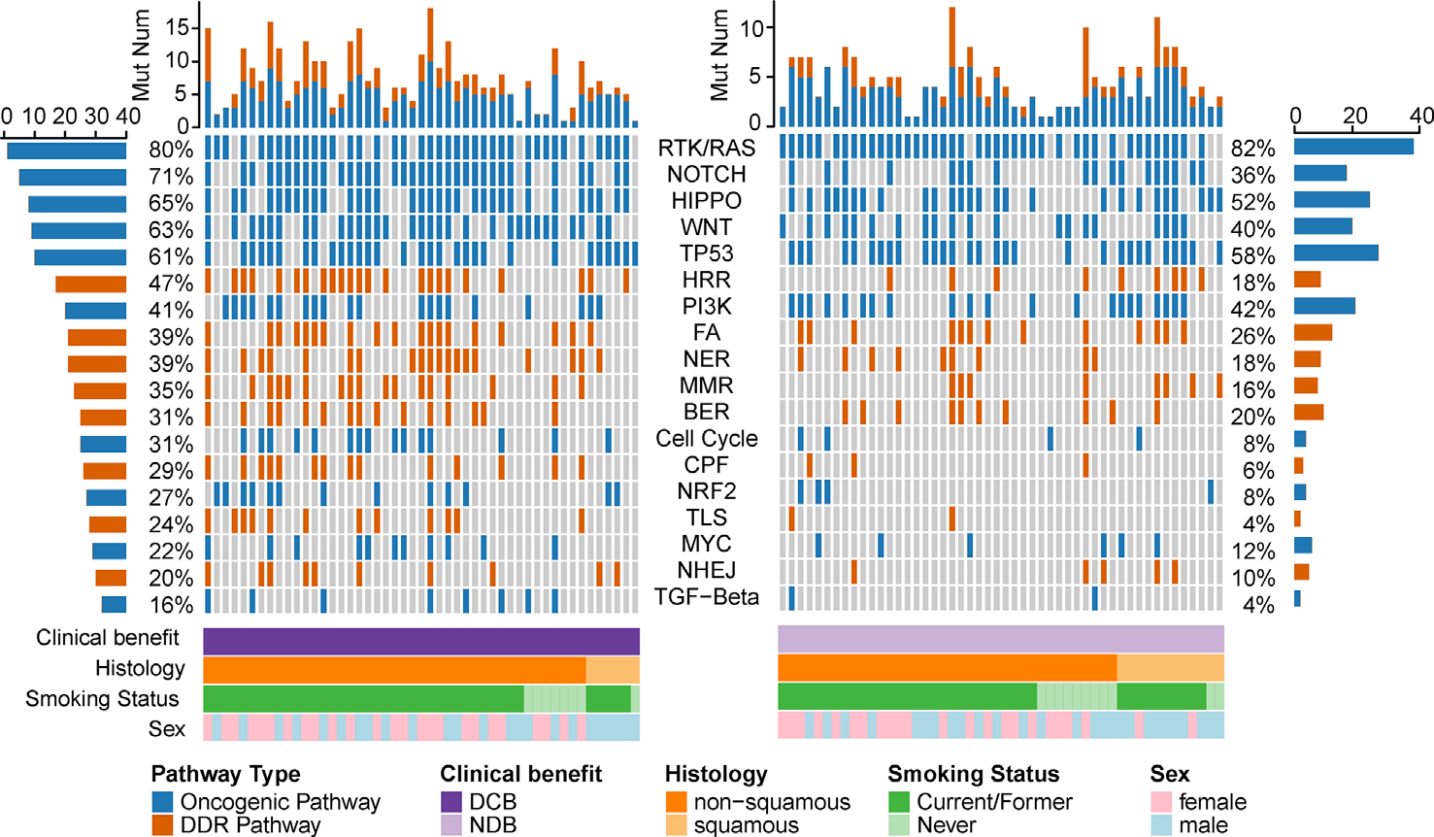
Pathway alterations in NSCLC with or without efficacious immunotherapy. Stacked plot showed the number of mutated pathway (histogram, top). Clinical characters are listed at the bottom of the figure. The prevalence of mutated pathways was calculated at left (in DCB group) and right (in NDB group). Orange represents the oncogenic pathway and blue stands for the DDR pathway.

We collected WES data from 106 NSCLC patients treated with immunotherapy derived from published data as the discovery cohort (15, 19). Of these patients, 51 (48.1%) achieved durable clinical benefit and 55 (51.9%) had no response to the immunotherapy. The clinical features and efficacy outcomes were summarized in **Table 1**. To unravel the determinants of response to immunotherapy on pathway levels, we mapped all mutated genes to 10 oncogenic signaling pathways and eight DDR pathways (**Supplementary Table 2**, **Figure 1**). A tumor sample was considered as altered in a given pathway if one or more genes in this pathway contained non-synonymous mutations. Data from 99 patients succeeded to map to pathways that were further analyzed. The most frequently altered pathway was RTK/RAS in both DCB and NDB group (80 *vs* 82%), followed by HIPPO pathway (65% in DCB *vs* 52% in NDB). Notably, 71% patients with DCB had alteration in NOTCH pathway while only 36% in NDB group (p = 0.005). This was consistent with previous study that NOTCH signaling correlated with better ICI efficacy (17). More DDR-related pathways were altered in DCB. Co-mutations in DDR pathways (hereafter referred as co-DDR) have been reported to a better clinical benefit to ICI by Wang et al. (18), we also detected a higher prevalence of co-DDR pathway alteration in DCB group (69.3 *vs* 30%, p = 0.0002). Taken together, alterations on pathway level can reflect the divergent response in immunotherapy.

**Table 1:** Clinical characteristics of discovery cohort.

	Total	DCB	NDB	P-value
**Histology**				
Squamous	87 (82.1%)	44 (86.3%)	43 (78.2%)	0.32
Non-squamous	19 (17.9%)	7 (13.7%)	12 (21.8%)	
**Sex**				
Female	54 (50.9%)	26 (51.0%)	28 (50.9%)	1
Male	52 (49.1%)	25 (49.0%)	27 (49.1%)	
**Smoking Status**				
Current/Former	85 (80.2%)	43 (84.3%)	42 (76.4%)	0.34
Never	21 (19.8%)	8 (15.7%)	13 (23.6%)	
**PD L1 expression**				
Strong	18 (17.0%)	14 (27.5%)	4 (7.3%)	0.045
Weak	48 (45.3%)	21 (41.2%)	27 (49.1%)	
Negative	31 (29.2%)	12 (23.5%)	19 (34.5%)	
Unknown	9 (8.5%)	4 (7.84%)	5 (9.1%)	
**Best Overall Response**				
CR/PR	34 (32.1%)	34 (66.7%)	0 (0.0%)	<0.001
PD/NE	36 (34.0%)	0 (0.0%)	36 (65.5%)	
SD	36 (34.0%)	17 (33.3%)	19 (34.5%)	
**Treatment**				
PD-1 blockade	31 (29.2%)	14 (27.5s%)	17 (30.9%)	0.83
PD-1 plus CTLA-4 blockade	75 (70.8%)	37 (72.5%)	38 (69.1%)	

CR, complete response; CTLA-4, cytotoxic T-cell lymphocyte-4; DCB, durable clinical benefit; NDB, no durable benefit; NE, not evaluable; PD, progressive disease; PD-1, programmed cell death-1; PR, partial response; SD, stable disease.

### Identification of NOTCH Co-occurring Pathways to Distinguish ICI Efficacy

**Figure 2:**
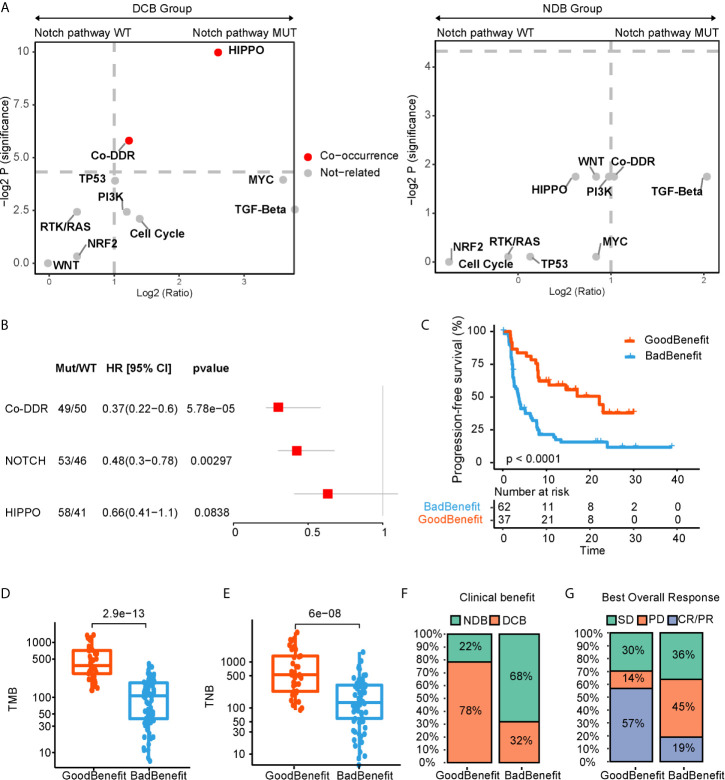
NOTCH co-occurring pathways that contributed to ICI efficacy. **(A)** log2 (odds ratio) and -log2 (p value) for enrichment of altered pathways co-occurred with and without NOTCH pathway. **(B)** Forest plot displaying univariate cox regression analyses for PFS of individually considered pathway. **(C)** Kaplan-Meier survival curves for PFS comparing “GoodBenefit” *versus* “BadBenefit.” **(D, E)** Boxplot of TMB **(D)** and TNB **(E)** in “GoodBenefit” *versus* “BadBenefit.” **(F, G)** Percentage of patients with clinical benefit evaluation **(F)** and objective response **(G)**.

Given that single pathway fails to adequately reveal the response of ICI, we applied a co-occurrence strategy to investigate how the NOTCH co-occurring pathways impacted the ICI efficacy. Comparing to NDB group, we identified the HIPPO and/or co-DDR pathway co-occurring with NOTCH pathway, which displayed a better clinical benefit (**Figure 2A**). After testing in univariate and multivariate cox regression for progressive free survival (PFS), only co-occurrence of NOTCH and co-DDR pathway proved to be an independently protective factor for PFS (**Figure 2B**, **Supplementary Figure 1**). Thus, 99 patients were further stratified into two groups according to the co-occurrent alteration of NOTCH and co-DDR pathways (hereafter referred as NOTCH+/co-DDR+). Altered genes for each pathway of NOCTH+/co-DDR+ were shown in **Supplementary Figure 2**. We defined the patients with NOTCH+/co-DDR+ as “GoodBenefit” (37 patients) and others were “BadBenefit” (62 patients). Patients grouped as “GoodBenefit” showed a longer PFS (22.1 *vs* 3.6 months, p < 0.0001, **Figure 2C**). Notably, co-occurrence of NOTCH+/co-DDR+ showed more prolonged PFS than single pathway (alteration either in NOTCH or co-DDR pathway) (**Supplementary Figure 3A**). In the NOTCH+/co-DDR+ group, we also detected higher tumor mutation burden (TMB, **Figure 2D**), higher tumor neoantigen burden (TNB, **Figure 2E**), more durable clinical benefit (p < 0.001, **Figure 2F**), and more improved objective response (p < 0.001, **Figure 2G**). These results were also observed when we stratified patients into the following three groups: CoPath (NOTCH+/co-DDR+) *versus* SinglePath (alterations in NOTCH or at least two DDR pathway) *versus* wild type (**Supplementary Figures 3B–E**). In summary, we identified NOTCH co-occurring with co-DDR pathways reflecting a better immunotherapy efficacy.

### Independent Validation of the Model in Two Cohorts

To evaluate whether the co-occurrence of NOTCH+/co-DDR+ could serve as a potential predictor of immunotherapy efficacy, we crossed-validated our findings using two independent cohorts who received ICI therapies.

**Figure 3:**
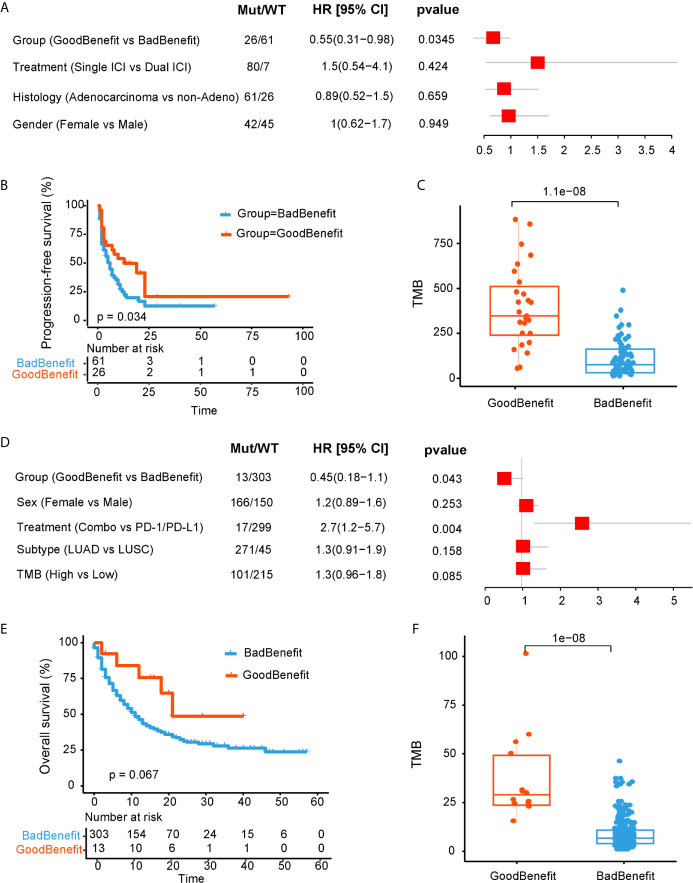
Validation of NOTCH/co-DDR co-occurring pathways to ICI efficacy in Anagnostou et al. cohort **(A–C)** and MSKCC cohort **(D–F)**. **(A)** Forest plot displaying univariate cox regression analyses for PFS of individually considered pathway for Anagnostou et al. dataset. **(B)** Kaplan-Meier survival curves for PFS comparing “GoodBenefit” *versus* “BadBenefit.” **(C)** Boxplot of TMB in “GoodBenefit” *versus* “BadBenefit.” **(D)** Forest plot displaying univariate cox regression analyses for OS of individually considered pathway for MSKCC data. **(E)** Kaplan-Meier survival curves for OS comparing “GoodBenefit” *versus* “BadBenefit.” **(F)** Boxplot of TMB in “GoodBenefit” *versus* “BadBenefit.”.

One validated dataset was from Anagnostou and colleagues (20), 87 NSCLC patients with definitive response evaluation were obtained. Using our stratification criteria, 26 patients were grouped into “GoodBenefit,” where 61 were classified as “BadBenefit.” Utilizing the univariate cox regression for PFS, “GoodBenefit” group showed a decreased risk of PFS (**Figure 3A**). Comparing to the group of “BadBenefit,” patients with co-existing pathways had longer PFS (13 *vs* 6 months, p = 0.034, HR, 0.55, 95% CI, 0.31–0.96) (**Figure 3B**). As expected, the TMB was significantly higher in “GoodBenefit” group than “BadBenefit” group (**Figure 3C**). When Comparing among the three groups (NOTCH+/co-DDR+ *versus* NOTCH or co-DDR occurrence *versus* Wildtype), co-occurrence of NOTCH/co-DDR also exhibited superior survival outcomes and the highest TMB (**Supplementary Figures 4A, B**).

For another independent cohort from MSKCC, 1,661 patients received immunotherapy by targeted sequencing, we retained the 316 NSCLC tissue [271 lung adenocarcinoma (LUAD) and 45 Lung squamous cell carcinoma (LUSC)] for further analyses (21). Co-occurrence of NOTCH+/co-DDR+ was found act as a protective factor (**Figure 3D**). Overall survival in co-occurrence group was prolonged, although did not reached the statistical significance (21 *vs* 11 months, p = 0.067, HR, 0.45, 95% CI, 0.181.1, **Figure 3E**). We speculated the limited genes from panel may underestimate the pathway alteration. In this cohort, targeted capture panel comprised of 341 and 410 genes, respectively, covering 10 oncogenic pathways and seven DDR pathways without TLS pathway. In addition, the TMB was significantly higher in “GoodBenefit” group (**Figure 3F**). When compared among the three groups, the co-occurrent group still performed improved OS prognostication and higher TMB (**Supplementary Figures 4C, D**).

### Genomic Characteristics of NOTCH/co-DDR Co-occurrence in TCGA Cohort

**Figure 4:**
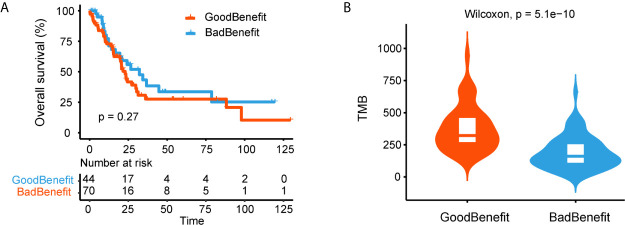
Genomic characteristics of NOTCH/co-DDR co-occurring pathways in TCGA cohort. **(A)** Kaplan-Meier survival curves for OS comparing “GoodBenefit” *versus* “BadBenefit.” **(B)** Violin plot of TMB between “GoodBenefit” *versus* “BadBenefit.”.

We next explored the genomic characteristics in TCGA cohort according to our stratification criterion. One thousand twenty-six NSCLC WES data and clinical features were downloaded. All mutated genes were mapped to 18 canonical pathways. TMB in “GoodBenefit” group was significantly higher than “BadBenefit” group regardless of stage (**Supplementary Figure 5A**). Considering the predictor of NOTCH+/co-DDR+ was trained based on advanced NSCLC and without EGFR mutation. We filtered out the patients with early stage and EGFR alteration. Ultimately, 120 advanced NSCLC patients retained; 39.2% (47) patients were classified as “GoodBenefit” group while 60.8% (73) as “BadBenefit” group. This ratio of potential efficiency coincided with previous reports on NSCLC patients beneficial of ICIs delivery (26–29). In order to exclude NOTCH+/co-DDR+ as a potential prognostic factor, we performed the survival analyses in TCGA cohort treated with standard treatment. No significant difference of overall survival was detected between GoodBenefit *versus* BadBenefit group or among three groups (**Figure 4A**, **Supplementary Figure 5B**). This result demonstrated co-occurrent NOTCH+/co-DDR+ was not a prognostic factor *per se*, but can serve as predictor in condition of immunotherapy. Recent studies have shown the TMB was a predictor of the pathological response to immune checkpoint inhibitors treatment in advanced lung cancer patients, we next checked the TMB distribution in our groups. Comparing to wildtype, higher TMB was observed in “GoodBenefit” group (**Figure 4B**). In details, the TMB was significantly higher in co-occurrence group than those harbored single pathway (either NOCTH or co-DDR alteration) and wildtype (neither NOTCH nor co-DDR alteration) (**Supplementary Figure 5C**).

### Immunobiology Features of NOTCH/co-DDR Co-occurrence

**Figure 5:**
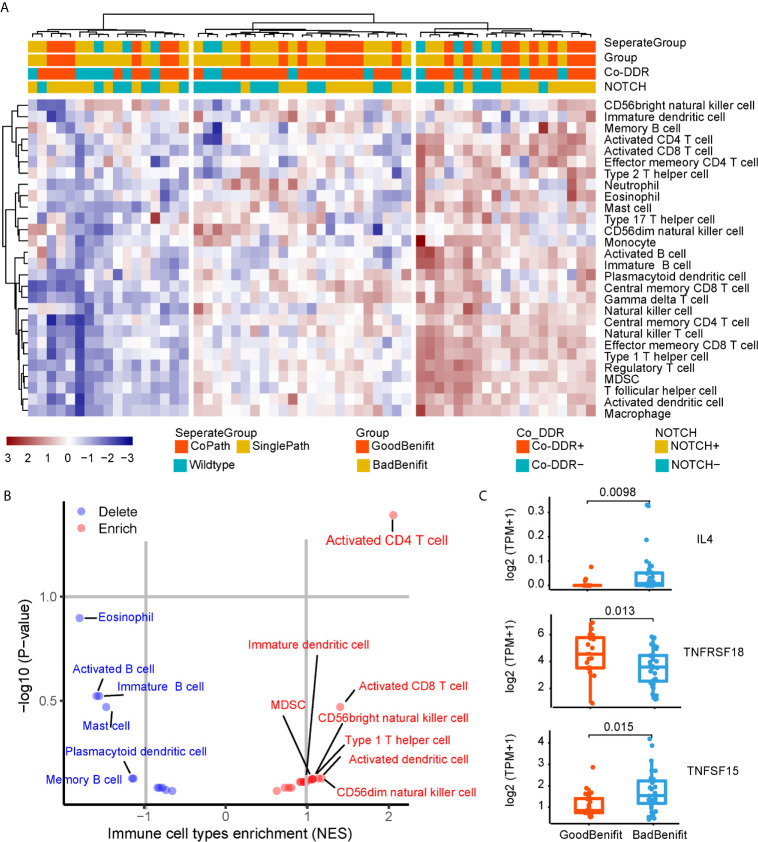
Immunobiology features of NOTCH/co-DDR co-occurrence. **(A)** Heatmap of immune infiltration in each patient. **(B)** Volcano plots for the enrichment (red) and depletion (blue) of immune cell types for GoodBenefit and BadBenefit. The expression levels of genes were first z-score normalized across all patients. Then we calculated the mean z-scores for each group and ranked in descending order. Based on the pre-ranked GSEA method, for each immune cell signature, we defined the q-value <10% and NES >0 as the enrichment, while the q-value <10% and NES <0 as the depletion. Immune cells with absolute NES greater than 1 were shown. **(C)** Normalized express of immune-related genes between Goodbenefit *versus* Badbenefit.

To further explore the immunobiology features of NOTCH+/co-DDR+ co-occurrence, we obtained 59 of 120 patients from TCGA whose RNA-seq data were available. The single sample gene set enrichment analysis (ssGSEA) method was employed to deconvolute the relative abundance of each immune cell type. By unsupervised clustering, we classified the 59 NSCLC into three clusters [immune-high (n = 27), immune-intermediate (n = 15), and immune-low (n = 17), **Figure 5A**]. Patients with NOTCH+/co-DDR+ co-occurrence had no preference to specific immune group (immune-high 14/27 *vs* immune-intermediate 3/15 *vs* immune-low 6/17, p = 0.12). Of note, CD4+ T cell were enriched in NOTCH+/co-DDR+ group (**Figure 5B**). We also found Interleukin 4 (IL-4), a quintessential T helper type 2 (Th2) cytokine produced by CD4+ T cells, expressed lower in “GoodBenefit” group (**Figure 5C**). TNFRSF18 was up-regulated in “GoodBenefit” group (**Figure 5C**), playing a role in promoting T effector cell activity by inducing proliferation and supporting survival in T cells, while also suppressing Treg activity (30). Another Tumor necrosis factor superfamily, TNFSF15 was downregulated in “GoodBenefit” group. TNFSF15 has been reported being an inhibitor of endothelial cell growth (31). Functional enrichment analyses revealed “GoodBenefit” group up-regulated growth factor receptor pathway, down-regulated DNA repair pathways and immune response pathways, this tumor context may contribute to the clinical benefit of ICI (**Supplementary Figure 6**).

## Discussion

In this study, we demonstrated the co-occurrence of NOTCH and co-DDR pathways can predict the superior survival outcomes after immunotherapy in advanced NSCLC. This phenomenon was first identified in one combined cohort and validated across another two independent cohorts. All the data we analyzed was performed WES, except one validated cohort from MSKCC (targeted sequencing). Using WES data was an advantage because it can cover enough genes of the pathway, overcoming the deficiency of targeted sequencing. To the best of our knowledge, this is the first report focusing on co-occurrence event on pathway level to explore the determinants of response to ICIs.

To explore whether there were dominant gene mutations in the co-occurring pathway, we depicted the co-occurring event on gene level involved in NOTCH and DDR pathways (**Supplementary Figure 1**). It can be observed that more alterations occurred in DCB group than NDB group. However, the overall mutation incidence on gene level was quite low. The most frequently mutated genes in NOTCH pathway were NOTCH1 (7%), NOTCH2 (7%), and JAG2 (7%). Meanwhile, low mutation rates of genes were observed in DDR pathway. Taken together, our data suggested that different genes converging to the co-occurring pathways effect the immunotherapy effect, rather than the specific gene of co-occurrence drives.

Although a single pathway of NOTCH or co-DDR has been reported as a predictor to ICI, we revealed co-occurrent NOTCH+/co-DDR+ pathway preferred in DCB and was more predictive to patients beneficial of ICIs delivery. In the first combined cohort, patients carrying co-mutations in NOTCH and co-DDR pathways had significantly longer PFS as compared to the SinglePath (alterations in NOTCH or at least two DDR pathway) or wild type, and the TMB was gradually decreasing. As for the validation dataset of cohort 2, we also observed the longer PFS in CoPath group than SinglePath, but there was no significant different in SinglePath *versus* Wild type. Thus, we speculated alteration in single pathway of NOTCH or co-DDR was incomplete correlate of immunotherapy.

Patients carrying alterations of NOTCH+/co-DDR+ pathway associated with increased TMB, TNB, and more infiltration of CD4+ T cell. Previous study has revealed that alterations in co-DDR pathway appeared to higher TMB and TNB (18). Since a higher mutation and neoantigen load could induce T cell-mediated antitumor response (32), we speculated that alterations in co-DDR contributed to more mutation and neoepitope, and further increased the likelihood of recognition by T cell. NOTCH signaling pathway has been reported as a pivotal role in regulating T cell modulation, differentiation, and activation (33, 34). In addition, previous study also reported NOTCH can control the fate of various T cell types, including Th1, Th2, and the regulatory T cells (35). In our result, we found co-occurrence of NOTCH/co-DDR tended to infiltrate more CD4+ T cell, but down-regulated the cytokine of Th2, suggesting more CD4 T cell that not secrete Th2 exist in NOTCH/co-DDR group. Moreover, recent studies have shown that NOTCH signaling is required for optimal T-cell-mediated anti-tumor immunity (36). Collectively, one potential explanation may be alteration of co-DDR pathway cause more mutations producing immunogenic neoantigens that are recognized and targeted by T cells and NOTCH pathway mediate more infiltration of CD4+ T cells and enhance effector T-cell activity. However, the underlying mechanism of co-ordination between NOTCH and co-DDR pathways and how these pathways shape together the microenvironment suit for immunotherapy should be further investigated in the future. Expanded data and more experiments are warranted to reveal the mechanism on how these pathways cross-talk to determine a better response to immunotherapy.

In conclusion, preliminary data from three cohorts showed evidence that co-mutations in NOTCH and co-DDR pathways indicated better immunotherapy efficacy. This genomic marker provided a new dimension to predict the superior survival outcomes in response to immunotherapy.

## Data Availability Statement

The original contributions presented in the study are included in the article/**Supplementary Material**. Further inquiries can be directed to the corresponding authors.

## Author Contributions

CC, CS, and ZZ conceived and designed the present study. ZZ, YYG, XS, and JB contributed equally to this work. ZZ and CC wrote the manuscript. ZZ, YYG, and XS collected and interpreted data. JB and WG performed the data analysis. JM, JL, and XX gave material support. JH, BZ, and YFG helped in improving the language and correcting grammar mistakes. All authors contributed to the article and approved the submitted version.

## Funding

China Postdoctoral Science Foundation funded project (Grant #2014T70977). Hubei Province National Natural Science Foundation of China (Grant #2018CFB733).

## Conflict of Interest

The authors declare that the research was conducted in the absence of any commercial or financial relationships that could be construed as a potential conflict of interest.
