# Supplementary material for: Co-Occurring Alteration of NOTCH and DDR Pathways Serves as Novel Predictor to Efficacious Immunotherapy in NSCLC
Source: Front Oncol. 2021 Apr 22;11:659321. doi: 10.3389/fonc.2021.659321 (PMC8100434; doi:10.3389/fonc.2021.659321)
Supplement: Supplementary file 1 [file DataSheet_1.pdf]

Supplementary Material

1 Supplementary Figures

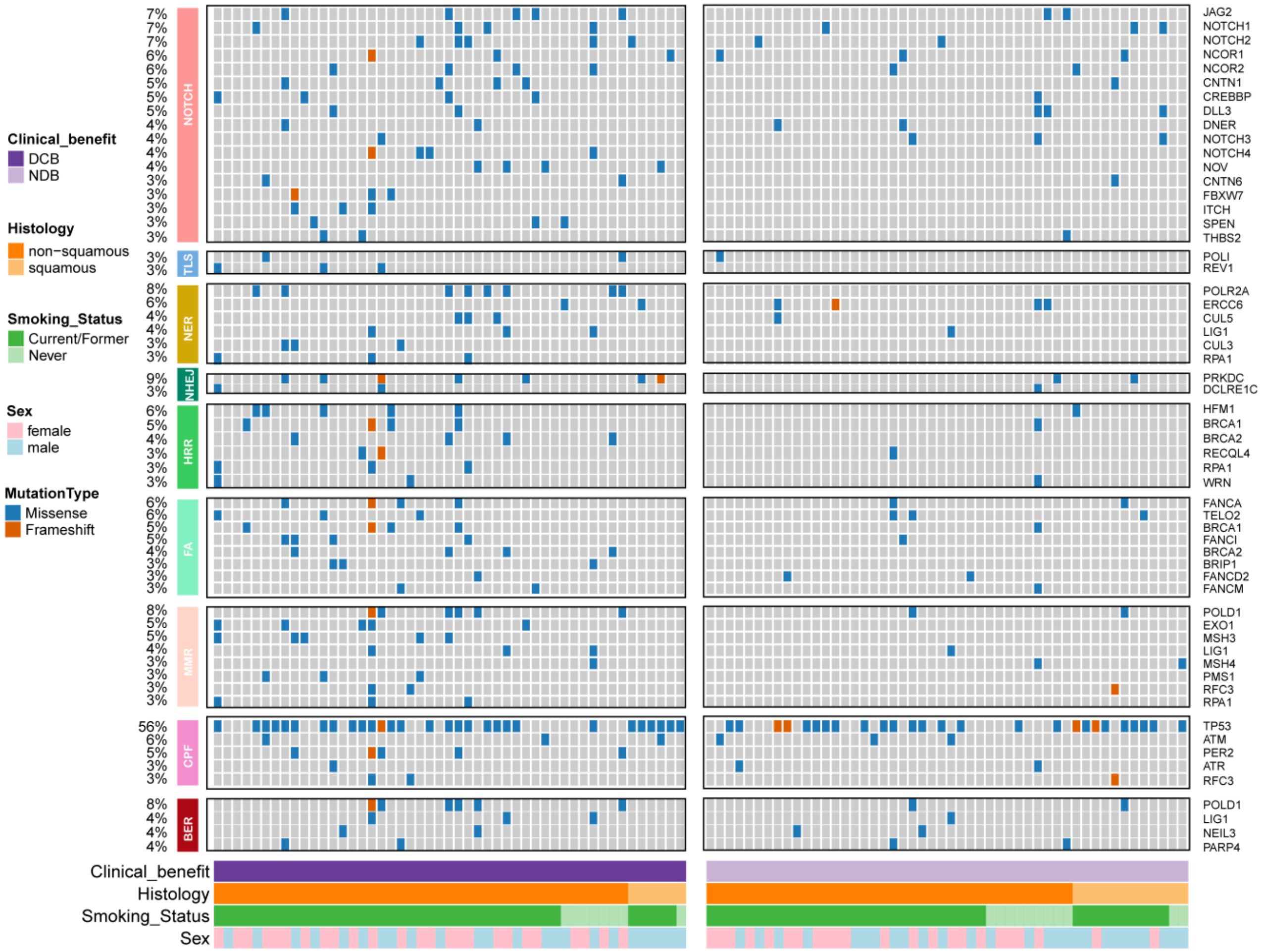

Supplementary Figure 1. Heatmap of gene alterations in NOTCH and DDR pathways. Patients' features including clinical benefit after immunotherapy, histology, smoking status and sex were listed. Genes with more than 3% incidence were showed.

A

Univariable analysis	Hazard ratio [95% CI]	pvalue
Group(BadBenefit vs GoodBenefit)	0.25(0.14-0.45)	6.68E-07
Histology(non-squamous vs squamous)	1.3(0.67-2.5)	0.452
Sex(Female vs Male)	1.4(0.86-2.3)	0.169
Smoking_Status(Current/Former vs Never)	1.9(1-3.4)	0.0469
PD L1 expression(Negative vs Weak or Strong)	0.44(0.19-0.99)	0.0752
Treatment(PD-1 blockade vs PD-1 plus CTLA-4 blockade)	0.73(0.43-1.3)	0.272

B

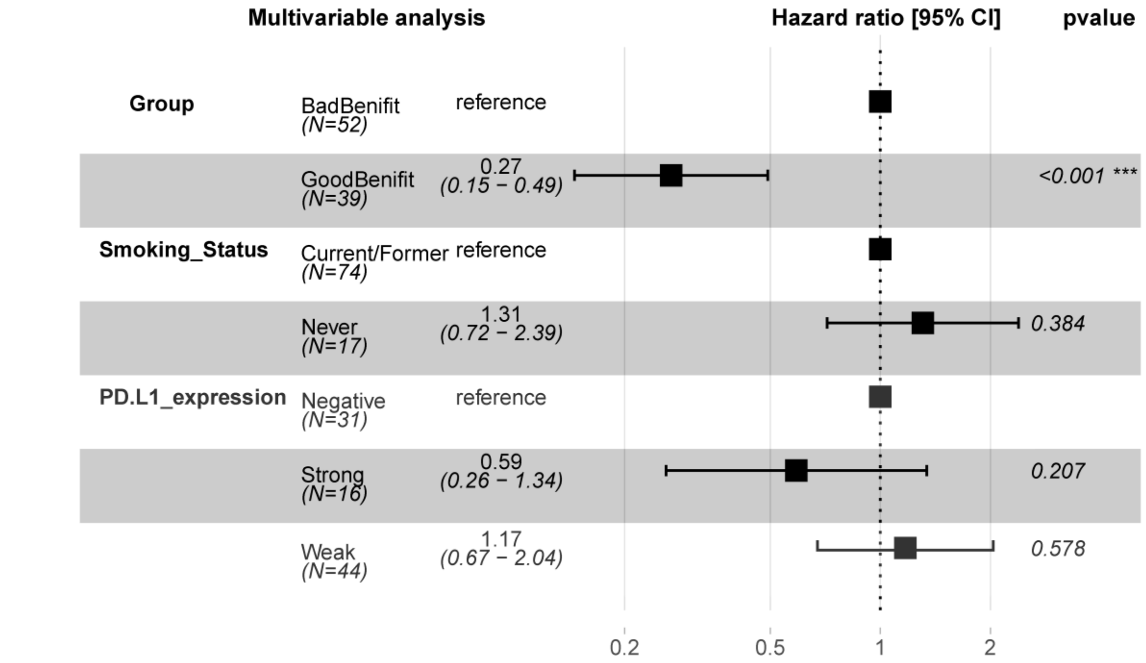

Supplementary Figure 2. Clinicopathology and co-occurring pathway contributed to ICI efficacy. **(A)** Univariate analysis was performed by cox regression to determine significant predictors for disease progression after immunotherapy. **(B)** Forest plot displayed multivariable cox regression including the factors with p values less than 0.1 by univariate analysis.

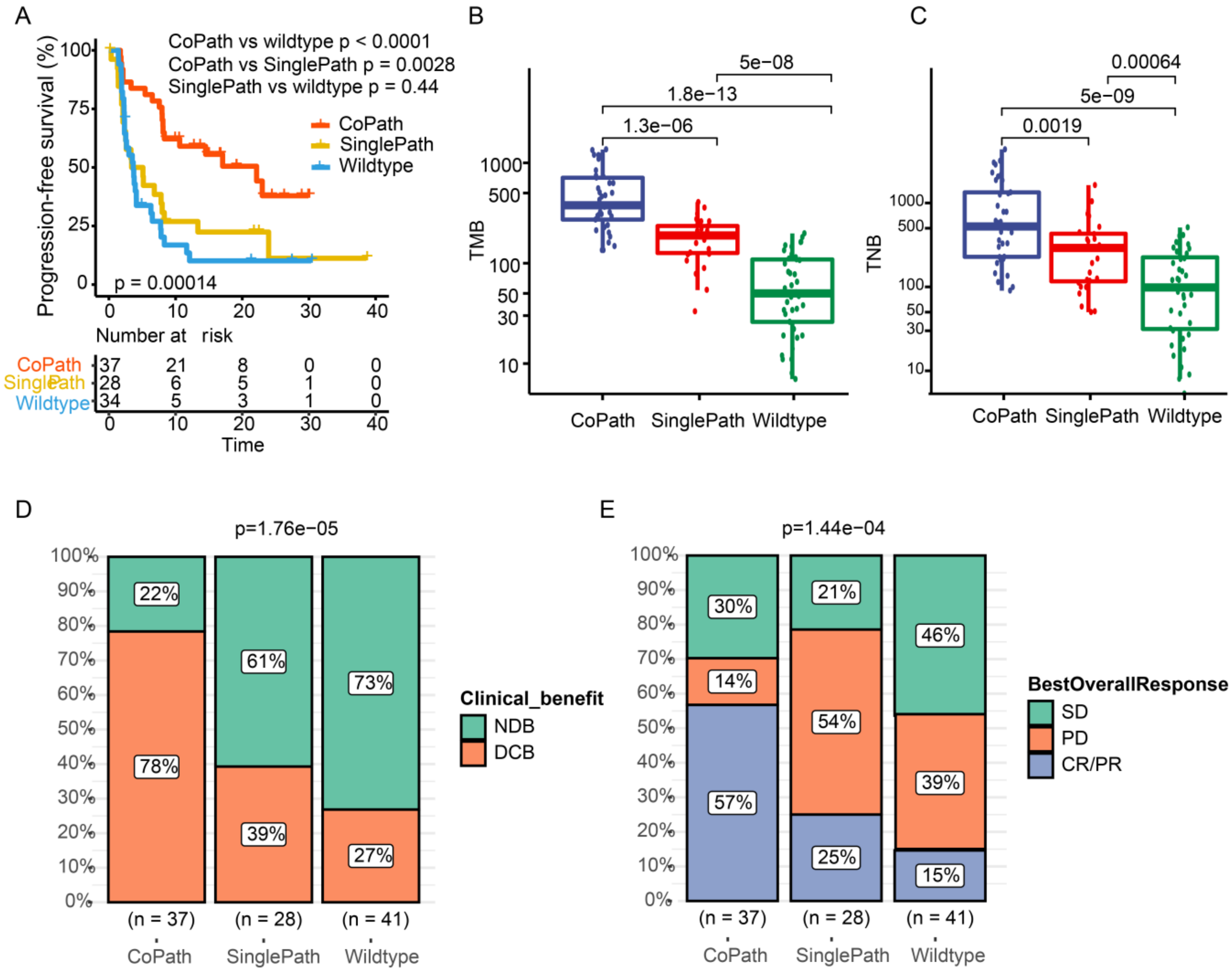

Supplementary Figure 3. NOTCH+/co-DDR+ co-occurring pathways that contributed to ICI efficacy in discovery cohort. **(A)** Kaplan-Meier survival curves for PFS comparing CoPath (NOTCH+/co-DDR+) versus SinglePath (NOTCH or co-DDR occurrence) versus Wildtype. **(B-C)**

Boxplot of TMB **(B)** and TNB **(C)** in CoPath versus SinglePath versus Wildtype. **(D-E)** Percentage of patients with clinical benefit evaluation **(D)** and objective response **(E)**.

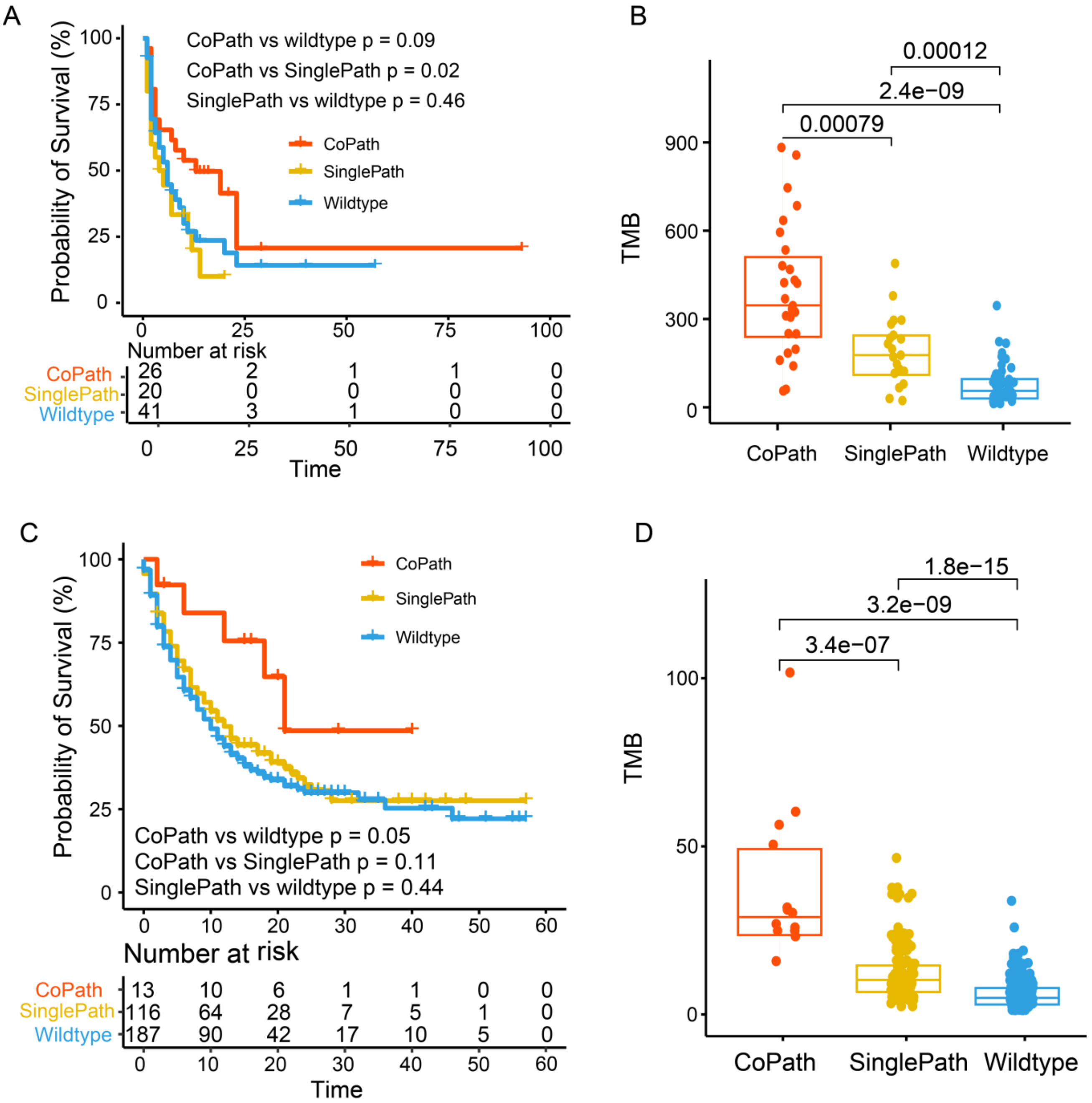

Supplementary Figure 4. Validation of NOTCH+/co-DDR+ co-occurring pathways to ICI efficacy in Anagnostou *et al.* cohort **(A-B)** and MSKCC cohort **(C-D)** among three groups. **(A)** Kaplan-Meier survival curves for PFS comparing CoPath (NOTCH+/co-DDR+) versus SinglePath (NOTCH or co-DDR occurrence) versus Wildtype. **(B)** Boxplot of TMB in CoPath versus SinglePath versus Wildtype. **(C)** Kaplan-Meier survival curves for OS comparing NOTCH/co-DDR co-occurrence

versus NOTCH or co-DDR occurrence versus Wildtype. (D) Boxplot of TMB in CoPath versus SinglePath versus Wildtype.

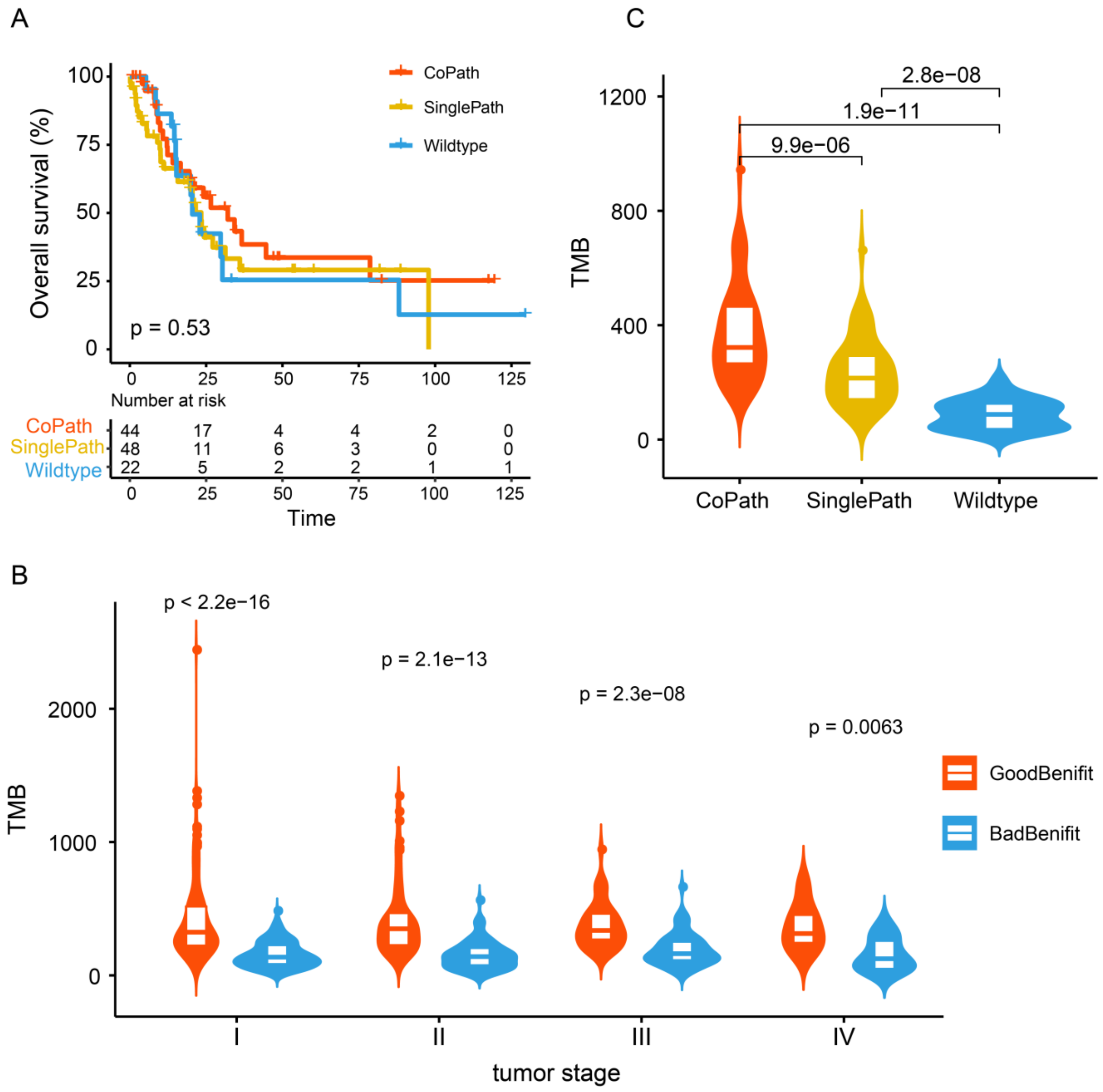

Supplementary Figure 5. Genomic characteristics of NOTCH+/co-DDR+ co-occurring pathways in TCGA cohort among three groups. **(A)** Kaplan-Meier survival curves for OS comparing CoPath

versus SinglePath versus Wildtype. **(B)** The TMB distribution of ‘GoodBenefit‘ versus ‘BadBenefit’ in different stage. **(C)** Violin plot of TMB among CoPath versus SinglePath versus Wildtype.

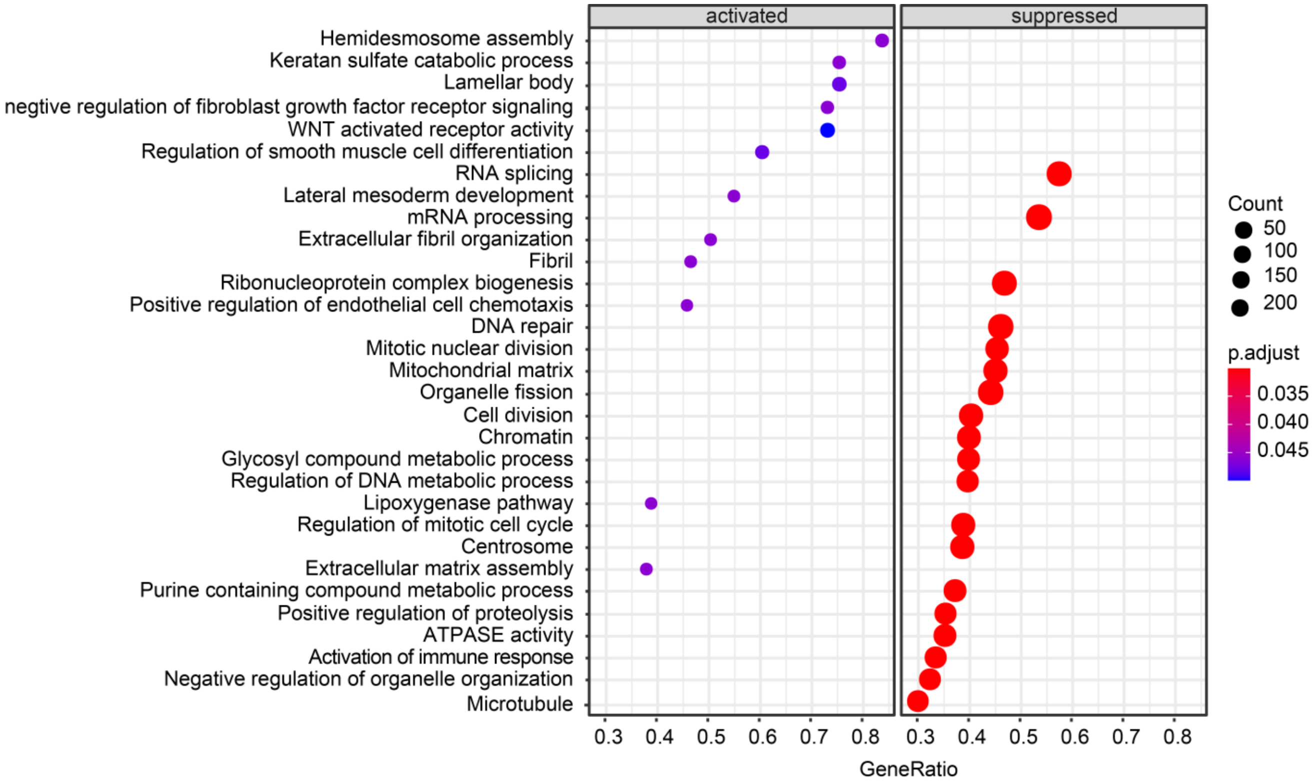

Supplementary Figure 6. Enrichment analysis of GO terms by genes between ‘GoodBenefit‘ versus ‘BadBenefit’.
